# Correlation of two-photon *in vivo* imaging and FIB/SEM microscopy

**DOI:** 10.1111/jmi.12231

**Published:** 2015-03-18

**Authors:** L Blazquez-Llorca, E Hummel, H Zimmerman, C Zou, S Burgold, J Rietdorf, J Herms

**Affiliations:** * Center for Neuropathology and Prion Research (ZNP) and German Center for Neurodegenerative Diseases (DZNE) – site Munich, Ludwig-Maximilians-University MunichMunich, Germany; † Carl Zeiss MicroscopyMunich, Germany; ‡ Munich Cluster of Systems Neurology (SyNergy), Ludwig-Maximilians-UniversityMunich, Munich, Germany

**Keywords:** Dendritic spine, electron microscopy, green fluorescent protein, three-dimensional reconstruction

## Abstract

Advances in the understanding of brain functions are closely linked to the technical developments in microscopy. In this study, we describe a correlative microscopy technique that offers a possibility of combining two-photon *in vivo* imaging with focus ion beam/scanning electron microscope (FIB/SEM) techniques. Long-term two-photon *in vivo* imaging allows the visualization of functional interactions within the brain of a living organism over the time, and therefore, is emerging as a new tool for studying the dynamics of neurodegenerative diseases, such as Alzheimer’s disease. However, light microscopy has important limitations in revealing alterations occurring at the synaptic level and when this is required, electron microscopy is mandatory. FIB/SEM microscopy is a novel tool for three-dimensional high-resolution reconstructions, since it acquires automated serial images at ultrastructural level. Using FIB/SEM imaging, we observed, at 10 nm isotropic resolution, the same dendrites that were imaged *in vivo* over 9 days. Thus, we analyzed their ultrastructure and monitored the dynamics of the neuropil around them. We found that stable spines (present during the 9 days of imaging) formed typical asymmetric contacts with axons, whereas transient spines (present only during one day of imaging) did not form a synaptic contact. Our data suggest that the morphological classification that was assigned to a dendritic spine according to the *in vivo* images did not fit with its ultrastructural morphology. The correlative technique described herein is likely to open opportunities for unravelling the earlier unrecognized complexity of the nervous system.

**Lay Description:**

Neuroscience and the understanding of brain functions are closely linked to the technical advances in microscopy. In this study we performed a correlative microscopy technique that offers the possibility to combine 2 photon in vivo imaging and FIB/SEM microscopy. Long term 2 photon in vivo imaging allows the visualization of functional interactions within the brain of a living organism over the time, and therefore, is emerging as a new tool to study the dynamics of neurodegenerative diseases, such as Alzheimer’s disease. However, light microscopy has important limitations in revealing synapses that are the connections between neurons, and for this purpose, the electron microscopy is necessary. FIB/SEM microscopy is a novel tool for three-dimensional (3D) high resolution reconstructions since it acquires automated serial images at ultrastructural level. This correlative technique will open up new horizons and opportunities for unravelling the complexity of the nervous system.

## Introduction

Progress in the understanding of brain functions are closely linked to the technical advances in microscopy. Cajal’s prolific work using the Golgi staining method largely founded the field of modern neuroanatomy (Cajal, 1888). Structural interaction between different neurons via synapses could be illustrated for the first time using microscopy. The contact zones between the neurones became a major focus point of modern neuroscience. Ongoing advances in light and electron microcopy have enabled new experimental approaches to inspect the nervous system across multiple scales, confirming a critical notion of neuron theory: the presynaptic and the postsynaptic elements in both invertebrate and vertebrate nervous systems are physically separated (Robertson, 1953; Palada, 1954).

Development of two-photon (2P) microscopy combined with electrophysiology allows the visualization of functional interactions within the brain of a living organism (Denk *et al*., 1990; Denk & Svoboda, 1997). Because of its superior imaging capability of deeper penetration into brain tissue and efficient detection of emitted photons, this method becomes an increasingly inspiring tool for neurobiologists. 2P imaging is therefore emerging as a new tool to study the dynamics of neurodegenerative diseases, such as Parkinson’s or Alzheimer’s disease, i.e. plaque deposition (Burgold *et al*., 2011) or alterations of the dendritic spines illuminating the progression of the pathology (Bittner *et al*., 2010, 2012).

However, it is important to bear in mind that the magnification provided by light microscopy is rather low (e.g. connections between brain regions, synaptic nature of the dendritic spines), and has important limitations regarding the resolution of important structural elements of synapses. Focus ion beam (FIB) techniques have already given new and powerful insights into ultrastructure of brain tissue (Merchán-Pérez *et al*., 2009; Blazquez-Llorca *et al*., 2013). The quantification and measurement of synapses is a major goal in the study of brain organization. However, a major limitation of this approach is that obtaining long series of ultrathin sections is extremely time-consuming and difficult. The most common method employed to estimate synaptic density in the human brain is indirect, by counting at the light microscopic level the immunoreactive puncta, using synaptic markers (DeFelipe, 2010). Correlative microscopy offers a possibility of combining dynamic deep-in-tissue non-linear optical (NLO) (2P) imaging and ultrastructural resolution. Using focused ion beam milling and scanning electron microscope (SEM) imaging (FIB/SEM microscopy), we visualized and reconstructed the same dendrites at 10 nm isotropic resolution that were formerly imaged over 9 days with an NLO system. This new way of correlative imaging could potentially open up new horizons and opportunities for unravelling the previously unrecognized complexity of the nervous system.

## Material and methods

### Protocols

1. Cranial window surgery (see section Cranial window surgery).
2. 2-Photon *in vivo* imaging (see section 2P microscopy).
3. Perfusion (see section Perfusion and ‘Near-infrared branding’ (NIRB) technique).
4. Marking the overview fields in the brain inside the intact head by mean of the NIRB technique (see section Perfusion and NIRB technique).
5. Cutting thick section from the window region (see section Perfusion and NIRB technique)
6. Marking dendrites within the thick section using NIRB technique (see section Perfusion and NIRB technique).
7. Cutting thin sections (see section Perfusion and NIRB technique).
8. Osmication and embedding of the selected thin sections (see section Preparation of the tissue for electron microscopy).
9. Preparation for FIB/SEM (see FIB/SEM imaging: Image processing and analysis)
10. FIB/SEM imaging and image analysis (see FIB/SEM imaging: Image processing and analysis).

### Cranial window surgery

Three-month-old male mice (*n* = 4) expressing green fluorescent protein (GFP) under *Thy-1* promoter were used (GFP-M line; Feng *et al*., 2000). Mice were singly housed in standard cages (30 × 15 × 20 cm). All procedures were in accordance with an animal protocol approved by the University of Munich and the government of upper Bavaria (Az. 55.2–1–54–2531–188–09).

For *in vivo* imaging, a chronic cranial window was implanted as described (Fuhrmann *et al*., 2007; Holtmaat *et al*., 2009). The mice were anesthetized with an intraperitoneal injection of ketamine/xylazine (14 mg kg^−1^ body weight; WDT eG, Garbsen, Germany/Bayer Health Care, Bayer AG, Leverkusen, Germany). Additionally, dexamethasone (6 mg kg^−1^ body weight; Sigma-Aldrich, Saint Louis, Missouri, USA) was intraperitoneally administered immediately before surgery. Utilizing the open-skull preparation, a cranial window was implanted above the somatosensory cortex. A z-shaped holder was firmly attached to the skull for securing the head of the animal in the custom-made head holder and repositioning of the same imaging field during repetitive series. After surgery, mice received subcutaneously analgesic treatment with carprophen (7.5 mg kg^−1^ body weight; Pfizer, New York, NY, USA) and antibiotic treatment (cefotaxim 250 mg kg^−1^ body weight; Pharmore, Barcelona, Spain).

### 2P microscopy

**Figure 1:**
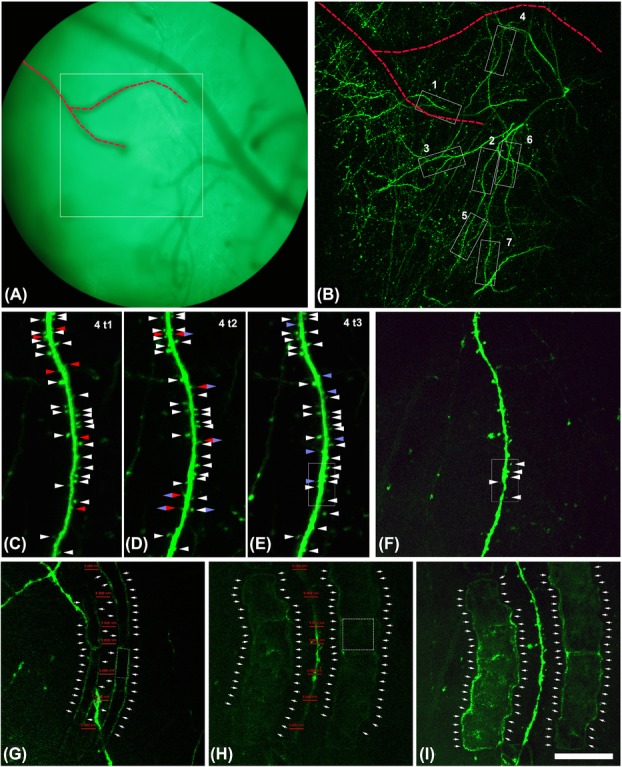
2P *in vivo* imaging of layer I dendrites of pyramidal neurons over time. (A) Blood vessel distribution was used in addition to coordinate setting to accurately relocate the same position for 2P imaging over time. Square in (A) surrounds the area that was imaged in (B). (B) Overview of the selected dendrites taken *in vivo* for the correlative approach. Stack of 100 images with a z-step of 3 μm. Note that the dashed red line indicates the same blood vessels in (A) and (B) that are useful to relocalize the dendrite 4. The same blood vessels are indicated in Figure2. C–E: Dendrite 4 *imaged in vivo* at different time points (1 week between *t*_1_ and *t*_2_; 1 day between *t*_1_ and *t*_2_). Stack of 20 images with a z-step of 1 μm. Note that white arrow heads point out spines that were present during the whole imaging period. Red arrow heads point out spines that will disappear in the next time point. Blue arrow heads point out spines that appear in this time point. Blue and red rhombus point out spines that appear in this time point but they are no longer present in the next time point. Blue and white rhombus points out spines that appear in this time point and that are present until the last imaging time point. (F) Dendrite 4 imaged *ex vivo* in the thick section before the laser marking. Note that the white rectangle present in (E) and (F) surround the dendritic segment that was further imaged and reconstructed with the FIB/SEM (see Fig.3). Stack of 20 images with a z-step of 1 μm. (G–H) Single plane of dendrite 4 relocated *ex vivo* in the thick section after the laser marking, 10 μm over the dendrite (G) and in the same focal plane of the dendrite (H). Marks are necessary to recongnize the region that has to be scanned with the FIB/SEM. Marks (single crop frame around 10 × 10 μm, dashed line cube in H) were made around the dendrite of interest in the central focal plane of the dendrite (around 5–10 μm far from the dendrite (H). Ten micrometers over this central plane other smaller marks (single crop frame around 10 × 5 μm, dashed line rectangle in G) were made resembling the profile of the dendrite (G). Note that red lines in G and H are located in the same position in both images and their size is 5 μm. Thus, it is observed how the marks made 10 μm over (G) resemble perfectly the shape of the dendrite located below (H). (I) Maximum intensity projection of dendrite 4. Stack of 20 images with a z-step of 1 μm. Laser marks are clearly visible. In (G–I), arrows point out the outer limit of the NIRB marks. Scale bar (in I): 134 μm in A, 89 μm in B, 19 μm in C–F, 26 μm in G–I.

Imaging began after a 3- to 4-week recovery period postsurgery, utilizing a ZEISS LSM 7MP setup (Zeiss, Germany) equipped with a MaiTai laser (Spectra Physics, Santa Clara, CA, USA). For *in vivo* imaging, mice were anesthetized by isoflurane, with individual sessions lasting for no longer than 60 min. The power of the laser was kept below 50 mW to avoid phototoxic effects. 2P excitation of GFP was performed at 880 nm, and a ZEISS 20× 1 NA water-immersion objective was used. Selected dendrites were imaged over 9 days. To find each time the same position, the motorized stage of the microscope was put in the corner limit left/front and this position was set as (0, 0). The coordinates of the brain regions that were imaged were established in reference to the (0, 0) point and noted down. Each imaging day, the same (0, 0) point was set and the same field of view was easily found by just entering its coordinates in the Zen Software. The variations finding the same position were ±100 μm. For fine relocation of the area of interest, the blood vessels were additionally used (Fig.1A). Between 7 and 10 dendrites located in layer I were selected for *in vivo* imaging in each animal (Fig.1B). The *in vivo* imaging was performed in three time points (*t*), the time between *t*_1_ and *t*_2_ was 1 week and between *t*_2_ and *t*_3_ 1 day, in this way we were able to distinguish between stable spines (persisting over at least 1 week) and transient spines (present only during 1 day; Figs.1C–E). Immediately after the last 2P *in vivo* imaging point (*t*_3_), the animals were transcardially perfused.

### Perfusion and NIRB technique

GFP-M mice were deeply anaesthetized with an intraperitoneal injection of ketamine/xylazine (14 mg kg^−1^ body weight; WDT/Bayer Health Care) and perfused transcardially at room temperature with PBS (0.1 M), followed by 2% paraformaldehyde and 2.5% glutaraldehyde in 0.12 M sodium phosphate buffer (PB), pH 7.4. After the perfusion, the head was cut off by cervical dislocation and the skin was removed and the remaining skull with the brain was left in the same fixative solution over 4 h.

**Figure 2:**
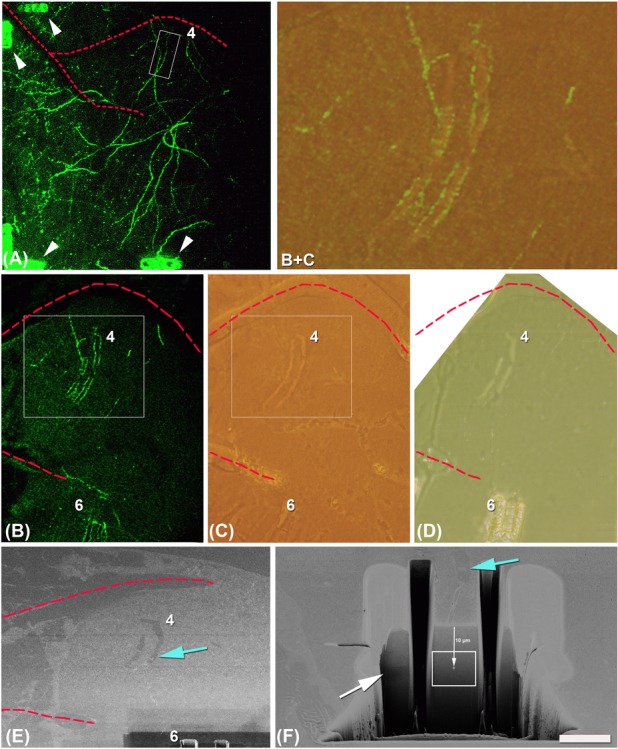
Laser marks are used for finding the dendrites of interest at the EM level. (A) Same overview of the selected dendrites for the correlative approach as in Figure1(B), taken *ex vivo* after the perfusion of the animal. Note that marks were made in some corners and borders of the overview (arrow heads) to facilitate the finding of the positions in the thick slice cut from the window region. (B) Single plane taken in the thin vibratome section (50 μm) showing two laser marks (around dendrites 4 and 6, the marks around dendrite 6 are only partially seen). The marks around dendrite 4 are the smaller ones performed 10 μm over the focal plane of the dendrite (as shown in Fig. 1G). (C) The same area as in (B) imaged postembedding. Laser marks could be easily identified. In this image, marks of dendrite 6 are not so clearly visible because the ones in focus belong to dendrite 4. (B and C) Area delimited in the rectangles in B and C and obtained after superposition of both images and decreasing the opacity of image B to 30%. Note that at the fluorescence level the most prominent part of the marks is the autofluorescence of the borders, however, at the light microcopy level after osmication the marks are visible as holes in the tissue and the borders are no longer so evident. (D) Last semi-thin section taken from the surface of the block once the smaller laser marks 10 μm over the focal plane of the dendrite 4 were reached. Note that around the marks of dendrite 6, a trench was previously opened with the FIB/SEM (as also seen in panel E). (E) Position recovery on the crossbeam. Laser marks are visible on the SEM image on the surface of the block. (F) Trench opened in front of the beginning of the mark and along the mark shown in (E) to free the region to image and avoid shadows. Note that serial FIB/SEM images were taken in the position and of the size indicated by the white rectangle. The centre of the images was established at 10 μm from the surface of the block and at the same z level of the bigger marks made at the focal plane of the dendrite 4 (with arrow; see Fig.1H). Blue arrow is pointing out exactly the same position of the superficial smaller mark (see Fig. 1G) in (E) and (F). Note that as in Figures1(A, B) the dashed red lines (in B–D) indicate the same blood vessels. Scale bar (in E): 91.4 μm in A, 50.8 μm in B–D, 18 μm in B + C, 43 μm in E, 7 μm in F.

Marking of the regions of interest was done in two steps with laser marks applied using the 2P-laser system according to the NIRB technique (Bishop *et al*., 2011). We used the crop tool (moving the crop area to make lines as many times as necessary) instead of the line scan for making the marks. The laser was tuned to *λ* = 800 nm and its power was increased to reach 300 mW at the back focal plane of the objective. Typically, we used 60–120 time series per crop area under visual control until appearance of autofluorescence. At the fluorescence level, the autofluorescence borders are the most evident part of the mark (Figs.1G–H and 2 B+C). Initially, the marking of the overview positions was done in the brain inside the intact head (Fig.2A); the size of the single crop frame was around 10 × 10 μm. Later, a thick section of the window region was cut (≈5 × 5 × 1 mm). This section was temporarily mounted in PBS, for laser branding, inside an imaging chamber custom-built over a slide and each individual dendrite was marked in this thick section (Figs.1G–I). Marks (single crop frame around 10 × 10 μm, see Fig.1I) were made around the dendrite of interest in its central focal plane (in 5–10 μm distance to the dendrite, Fig.1H). Ten micrometers over this central plane, other smaller marks (single crop frame around 10 × 5 μm, see Fig.1G) were made resembling the profile of the dendrite (Fig.1G). The laser brands could be easily identified around the dendrite of interest (Fig.1I). After the preparation of the tissue for electron microscopy, the fluorescence is lost and the marks are the references to find the dendrites of interest with the FIB/SEM.

Once the individual dendrites were marked within the thick slice, the tissue was resectioned on thinner slices of 50 μm. For this purpose, a high precision Leica Vibratome (Leica VT1200; Leica, Wetzlar, Germany) equipped with Shapiro blades was used. After the cutting, the sections were visualized again under the 2P microscope, to find slices with marked dendrites of interest (Fig.2B).

### Preparation of the tissue for electron microscopy

Selected 50 μm sections containing the dendrites of interest were osmicated for 1 h at room temperature in PB containing 1% OsO_4_ and 7% glucose. After washing in PB and one wash in 50% ethanol, the sections were, for 30 min, incubated in 1% uranyl acetate with 50% ethanol at 37°C, a step followed by dehydration and flat embedding in Araldite (DeFelipe & Fairén, 1993; Merchán-Pérez *et al*., 2009). After osmication, the fluorescence is lost but the marks around the dendrites remain visible as holes made in the tissue (Fig.2C). The shuffle and find function in the electron microscope software enables to recover the exact surface positions for FIB imaging.

Embedded sections were glued onto a blank Araldite block and trimmed. Semi-thin sections (1μm thick) were obtained by ultramicrotome Leica (EM UC 7; Leica) from the surface of the block until smaller marks located 10 μm over the dendrites of interest were reached (Fig.2D). Blocks containing the embedded tissue were then glued onto aluminium sample stubs using conductive carbon adhesive tabs (Electron Microscopy Sciences, Hatfield, PA, USA). All surfaces of the Araldite blocks, except for the surface to be studied (the top surface containing the sample), were covered with a colloidal silver paint (Electron Microscopy Sciences) to prevent charging artefacts. The stubs with the mounted blocks were then placed into a sputter coater (Emitech K575X, Quorum Emitech, Ashford, Kent, UK) and coated with platinum over 10 s, to facilitate charge dissipation. Importantly, the marks remained visible on the surface of the block processed with the FIB/SEM (Fig.2E).

### FIB/SEM imaging: Image processing and analysis

The ultrastructural three-dimensional (3D) study of these samples was carried out using a Cross beam AURIGA electron microscope. This instrument combines a high-resolution field-emission SEM column (Gemini column, Carl Zeiss) with a focused gallium ion beam, which permits the removal of few nanometre thin layers of material from the sample. As soon as one layer of material has been removed, the exposed surface of the sample is imaged by the SEM, using the backscattered electron detector. The sequential automated use of alternating FIB milling and SEM imaging allowed us to obtain long series of electron micrographs that represent 3D sample volumes of selected regions. Images of 2048 × 1536 pixels, at a resolution from 5.99 to 9.97 nm pixel^−1^ were taken; each individual electron micrograph therefore covered a field of view around 12.27 × 9.20 μm^2^. The layer of material milled by the FIB in each cycle (equivalent to section thickness) was 10 nm in all samples. The number of serial sections obtained for each sample varied between 135 and 1300. The milling current of the FIB ranged from 500 pA to 1 nA and the SEM was set from 1.8 to 2.0 kV acceleration potential.

**Figure 3:**
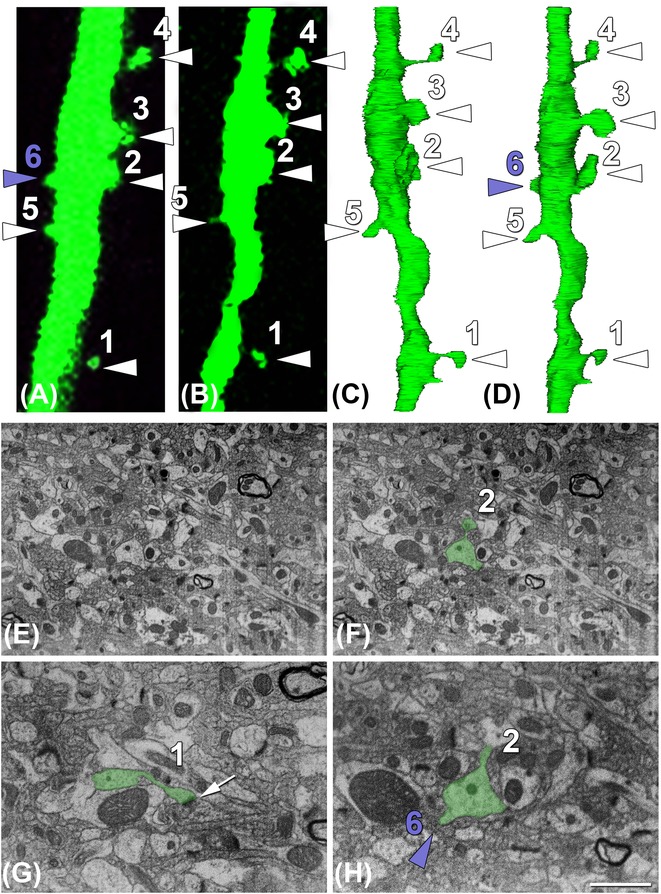
Correlative imaging of a segment of dendrite 4 using 2P and FIB Crossbeam imaging. (A, B) *In vivo* (last time point) and *ex vivo* imaging, respectively, of the segment of dendrite 4 that was further image with the FIB/SEM. (C, D) Three-dimensional reconstructions of the area of interest. Dendritic spines can be clearly localized. Note that the reconstruction in (C) resembles better the segment of the dendrite that was imaged *ex vivo* (B). (D) The same reconstruction as in (C) but slightly rotated so the dendritic spine number 6 that was present in the *in vivo* imaging (A) is also visible. (E, F) Example of one of the FIB/SEM images that were taken in the stack of 1300 serial sections before (E) and after (F) segmentation of the dendrite of interest (green). (G, H) Detail of two of the FIB/SEM images showing a synaptic (G; white arrow points out the synaptic contact) and a nonsynaptic dendritic spine (H; spine number 6). Note that the spines pointed out with a white arrow head (1–5) were present during the whole imaging period and thus, they correspond to ‘stable’ spines. All the stable spines established synapses with excitatory axons (see G in example). The transient spine number 6 (purple arrow head) that was only present in the last imaging time point did not establish synapses with any axon, thus it is a nonsynaptic dendritic spine (see H in example). Note that in some cases those dendritic spines defined as stubby at the fluorescent level (e.g. dendritic spine 2 in A, B) presented a differentiated and clear neck at the ultrastructural level (e.g. dendritic spine 2 in E, F). Scale bar (in H): 2 μm in (A–D), 2.7 μm in (E–F), 1.4 μm in (G, H).

For the alignment (registration) of the stack of images, we used the Fiji software (http://fiji.sc). We applied a rigid registration method (translation) to avoid the deformation of individual sections. After registration, the resulting stack was filtered and resized. We applied a Gaussian blur filter in Fiji to smooth the pixilation. Images in the stack were then scaled to one-half of their original size, to reduce the size of acquired data. Reconstruct Software1.1.0.0 (Fiala, 2005) was used to carry out the serial reconstruction of the dendrites and their 3D visualization (Fig.3).

## Results

### Methodological approach

The use of the described methodology allowed us to relocate and image dendritic structures with the FIB/SEM that were previously monitored *in vivo* using 2P microscopy. The here-described protocol is similar to that published by Maco *et al*. (2014, 2013) but with some differences. Previous works had already correlated 2P *in vivo* imaging and FIB/SEM microscopy with different approaches (Chen *et al*., 2011; Sonomura *et al*., 2013).

GFP-expressing dendrites of pyramidal neurons were imaged *in vivo* in the somatosensory cortex of adult mice with 2P microscopy (Figs.1A–E). The imaging was carried out through a glass cranial window as described elsewhere (Fuhrmann *et al*., 2007; Holtmaat *et al*., 2009). After imaging sessions, the animals were perfused with a fixative optimized to maintain the cellular ultrastructure.

After perfusion and prior to the removal of the brain from the cranial cavity, the region of interest was branded with fiducial marks (Fig.2A). To find the region of interest, we use the *x*, *y* coordinates of the overviews’ positions and the vasculature. The window region of the somatosensory cortex was then sliced in a thick section tangentially to the cortical surface, parallel to the imaging window and the focal plane of the 2P microscopy. The vasculature together with the marks made around the overview region provided landmarks that facilitated the relocation of fluorescent dendrites in the fixed sections (Fig.2A). Once the exact segments of dendrites were located, and orientated to the same position as seen in the living animal (Fig.1F), the 2P microscopy was used to brand fiducial marks around this region of interest (Figs.1G–H). We used the crop tool with sufficient laser power (see Methods) to burn stripes by repeating the crop both at the same focal plane of the dendrites (Fig.1H) and around 10 μm over them (Fig.1G). The marks were visible in the 2P microscope due to increased autofluorescence at the edges of the crop scans (Figs.1G–H). The thick section (around 1 mm) was resliced in 50 μm sections and those containing the dendrites of interest (Fig.2B) were selected and prepared for the EM. Marks themselves were clearly visible in the 50 μm resin-embedded sections (Fig.2C). Within the embedded material these marks were the only indication of the dendrites’ position, since at this stage any fluorescence had been completely lost.

Because of the visualization of the laser marks in the resin-embedded sections, they could be trimmed in the ultramicrotome with glass knives. Semi-thin sections were taken from the surface of the block until the marks located 10 μm over the dendrites of interest have been reached (Fig.2D).

Their visibility in the FIB/SEM (Fig.2E) was also used for aligning the block for final milling and imaging. The ion beam milled perpendicular to the block face, and thus perpendicular to the focal plane of the 2P microscope, and also to the plane of the brand marks. To free the imaging region and avoid shadows, a big trench of 25 × 25 × 25 μm^3^ was opened just in front of the beginning of the mark and another two smaller trenches were opened parallel and external to the borders of the mark (Fig.2F). As these first marks were about 10 μm over the dendrite of interest, images with their centres at 10 μm from the surface were taken with a frame size of approximately 12.27 × 9.20 μm (Fig.2F). The z-position of the dendrite was controlled by visualizing in the trench the coronal view of the bigger marks brand at the focal plane of the dendrite (Fig.2F white arrow). Stacks of around 1300 images were taken with a section thickness of 10 nm, thus summing up to total stack thickness of around 13 μm (logical size: 2048 × 1536 pixels, physical size: 12.27 × 9.20 μm and z-step: 10 μm). This stack could be obtained by running the FIB/SEM microscope autonomously overnight. If the same work is conducted with a transmission electron microscopy, most probably it would be impossible due to several reasons: low chance of collecting the whole series of ultra-thin sections, the minimum section thickness is 50 nm, the thickness is not homogeneous in different sections, the region of interest must be localized and imaged in all sections and further aligned.

3D reconstructions were performed using Reconstruct software (Fiala, 2005). Because of the superficial marks and those situated at the focal plane of the dendrite, the spatial position of the dendrite of interest was well defined. We found our candidates by looking at dendrites located in the centre of the images, with a shape of circle in each section and extending as a tube along the 13 μm stack. With these specific characteristics, only a few dendrites were our possible dendrite of interest (approximately, *n* = 7 in each stack). Manual rough reconstruction of all candidates was performed (segmentation of the dendrite was performed every five sections, thus is every 50 μm) and the dendrite of interest was found by judging its morphological appearance, shape resemblance at the fluorescent level. Once the dendrite of interest was found, a precise manual segmentation along the whole stack was performed in all sections (Fig.3).

### Correlation of dendritic spines visualized by 2P *in vivo* imaging and FIB/SEM microscopy

In a next step we proofed the usability of this technique looking at the ultrastructure of dendrites that were imaged *in vivo* and thus, have known track records of their dendritic spines. More specifically, we imaged dendrites in the somatosensory cortex of adult mice for 8 days, quantifying the gain and loss of spines. Using FIB/SEM microscopy, we analyzed the ultrastructure of spines and associated buttons that were followed *in vivo*. Those dendritic spines that we considered as ‘transient’ (Figs.1C–E and3) often lacked synapses, whereas spines that persisted for 8 days or more always had synapses. New spines normally had a filopodia-like morphology, with no clear head, evident at both the fluorescent microscopy and EM levels (Figs.1D, E and 3). Our data show that the spine growth precedes the synapse formation, as it was previously demonstrated by using 2P microscopy and transmission electron microscopy (Knott *et al*., 2006).

Interestingly, we found that occasionally the morphological characteristics of dendritic spines defined at the fluorescent microscopy level (e.g. mushroom, stubby and filopodia spines) did not fit with those revealed by ultrastructural observations. Interestingly, we found that spines defined as stubby at the fluorescent level (e.g. dendritic spine number 2, Figs.3A, B) frequently had a clear neck as well in the 3D reconstruction of the dendrite at the ultrastructural level (Fig.3). Thus, the results derived from morphological analyses of spines’ shape with fluorescence microscopy should be conducted with great caution, and especially, those made by *in vivo* 2P imaging, due to resolution limitations (see also Tønnesen *et al*., 2014).

## Discussion/conclusion

2P imaging enables scientists to study dynamic processes within living animals. Analysis of dendritic spines and their dynamics is of strong interest for many open questions in brain research. Combining 2P imaging with focused ion beam milling and imaging allows revealing large volume dynamics and correlating these at the ultrastructural level semi-automatically. Correlative microscopy offers the possibility to exactly relocalize the dendrites of interest and provide ultrastructural insights, which so far was only possible using laborious and time-consuming serial sectioning by mean of transmission electron microscopy. The combination of 2P and FIB/SEM methods avoids most of problems encountered using serial section and serial tomography methods. Missing wedges (due to the Crowther criterion) are no issue. Missing sections or differences of section thickness are not present. Large volumes can be analyzed with precision and high spatial resolution, because of the use of crossbeam systems. Time-consuming alignment procedures generally used in tomography are not needed, simple cross-correlation methods are sufficient to achieve a precise alignment of the data gathered. Taken together, the entire method makes up a new workflow, significantly reducing the time for the imaging, identification and reconstruction of previously imaged cell structures and thus, this approach opens the door for many investigations in neuroscience which are so far unanswered.
